# Identification of HN-1-Peptide Target in Head and Neck Squamous Cell Carcinoma Cells

**DOI:** 10.5402/2011/140316

**Published:** 2011-05-03

**Authors:** Jozsef Dudas, Christin Idler, Georg Sprinzl, Andreas Bernkop-Schnuerch, Herbert Riechelmann

**Affiliations:** ^1^Department of Otorhinolaryngology, Medical University Innsbruck, Anichstrasse 35, 6020 Innsbruck, Austria; ^2^Institute of Pharmacological Technology, Leopold-Franzens-University Innsbruck, Innrain 52, 6020 Innsbruck, Austria

## Abstract

The HN-1 module was previously reported to ensure efficient targeting of head and neck squamous cell carcinoma (HNSCC). Aim of this work was to indentify the target of HN-1. Targeting of HN-1 peptide was compared in normal epithelial cells (BEAS-2B) and in HNSCC tumor cells (SCC-25 and Detroit 562). Experimental, cell culture, cell polarity, and adhesion conditions were tested; structure models of peptides were created. Indeed, HN-1 was able to target HNSCC tumor cells in the previously published conditions. The targeting efficiency of immortalized normal epithelial cells was significantly lower. Nevertheless, in other experimental conditions the binding was less efficient and not specific. A scrambled sequence of HN-1, with altered order of amino acids showed even better targeting efficiency than HN-1. HN-1 was only uptaken in adherent cells, not in suspension. In conclusion, HN-1-peptide-targeting is not based on sequence specificity, but more on electrostatic interactions with the cell surface of the tumor cells.

## 1. Introduction

Promising new therapies for HNSCC require tumor-targeted approaches that afford tumor specificity and limited toxicity. Identification of tumor-specific peptides for targeted drug delivery into solid tumors rises an opportunity for tumor-specific delivery of therapeutic payloads [1]. 

The development of diverse peptide libraries over the past decade has ushered in the opportunity to identify small peptides that may not be as limited as the larger antibody predecessors [1]. Recently, several research groups developed peptide-based therapy ideas as options for tumor-specific targeting. Small peptides were often represented as tumor-specific targeting aids. As an example, Hsiao et al. developed a so-called Phage/peptide-29, which preferentially bound to integrin *α*v*β*6 rather than to other *α*v-associated integrins [2]. Peptide-29 significantly inhibited the proliferation of oral squamous cell carcinoma cells in 3D cell cultures. On human pathological sections, peptide-29 targeted oral cancer cells in a *α*v*β*6-dependent manner [2]. Nothelfer et al. also discuss an HNSCC affine peptide (HBP-1) derived from a phage display library [3]. Authors performed peptide immunohistochemistry of HNSCC tissue sections and exhibited tumor staining by HBP-1, whereas normal tissue remained negative. Sequence mutation and competition experiments revealed that the intrinsic RGD motif in combination with the intrinsic LXXL motif was responsible for the binding ability of HBP1. The RGDLXXL sequence within this peptide is known and indicates that binding occurs via the *α*v*β*6 integrin, which is exactly the same conclusion as the one of Hsiao et al. [2, 3]. 

In fact, peptide-targeting of HNSCC has already been published over ten years ago, by Hong and Clayman [1]. They have also used phage-display and found a novel peptide, HN-1, which internalized specifically to HNSCC cells. In 2009 Bao et al. combined the HN-1 peptide of Hong and Clayman [1] with a proteinkinase Cq (PKCq) inhibitory module, which has functioned as a specific PKCq inhibitor [4]. The efficiency and selectivity of HN1-PKCq combination was tested in HNSCC cells in vitro compared with oral epithelial cells. In contrast to a minimal labeling of normal epithelial cells (1.9%), the HN1-PKCq combination labeled up to 86.5% of the HNSCC cells [4]. This second study corroborated the findings of Hong and Clayman [1] and confirmed a HNSCC-specificity of HN-1, and its combination with PKCq-targeting reached effective antitumor actions [4]. 

Being HN-1 the only targeting peptide whose HNSCC-specificity has been independently proven by more research groups [1, 4], it was also chosen by us for a basis of a novel, targeted nanoparticle tumor therapy. The concept is to use selective targeting ligands on the surface of complex nanoparticles [5], whose binding and internalization specificity assures the selective delivery of cytostatic payload to tumor cells. In this study, in order to fit with our strict requirements for tumor selective targeting, the following aspects of HN-1 binding and internalization were investigated:

- sequence specificity of HN-1-mediated HNSCC-targeting,
- reproducibility of HN-1-mediated HNSCC-targeting,
- comparison of the targeting of immortalized epithelial cells (BEAS-2B), primary lingual HNSCC cells (SCC-25), and metastatic pharyngeal HNSCC cells,
- in which extent the localization of the original tumor of the cell line affects the targeting?
- influence of experimental conditions on the targeting by HN-1, usage of physiological conditions,
- identification of the target.

Our findings demonstrate that the published results of Hong and Clayman [1] and Bao et al. [4] are reproducible only in the same experimental conditions; HN-1 does not show an amino acid sequence-specific targeting, but it might be structure specific; HN-1 might bind to multiple targets on a surface of a polarized cell, but has no targeting function to suspended cells. HN-1 targets primary and metastatic tumor-derived cell lines and targets only at a very low efficiency immortalized, but not transformed cells. 

## 2. Materials and Methods

### 2.1. Cultured Cells

In this study, BEAS-2B immortalized bronchial epithelial cells [6], SCC-25 lingual squamous cell carcinoma cells [7], and Detroit 562 metastatic pharyngeal squamous cell carcinoma cells were used [8]. All of these cell lines were of commercial origin, and their culture conditions are summarized in Table 1. 

### 2.2. Peptides

In this study, the following peptides were used: HN-1 published by Hong and Clayman (2000) [1], a scrambled version of HN-1 was designed by Dr. Fritz Andreae (Pichem, Graz Austria), and another, with HN-1 not-related peptide sequence was also used and was labeled as “irrelevant”. The peptide sequences are summarized in Table 2. Peptides were synthesized by Pichem (Graz, Austria).

### 2.3. Structure and Folding Analysis of the Peptides

The peptide secondary structures and folding were predicted by PEP-Fold, an online resource for de novo peptide structure prediction [9]. PEP-Fold provided pdb (protein data bank) files with representation of macromolecular structure. Pdb files were visualized by a Deepview/Swiss-Pdbviewer [10–14]. Protein-protein interaction sites were predicted as described before [15].

### 2.4. Peptide-Binding in Cell Suspension

Cells were counted, and 1 × 10^6^ cells were incubated with 1 *μ*M peptides in PBS or in complete medium containing 10% fetal bovine serum for 10–30 minutes at room temperature [3], followed by centrifugation at 290 g, 4°C. Cells were washed two times with PBS and were then taken into Isoflow Sneath (Coulter, Vienna, Austria) for flow cytometry analysis. 

### 2.5. Incubation of Cells with Peptides

In Petri dishes, 0.5–1 × 10^6^ cells were plated and they were cultured routinely for 48 hours. Peptides were given to the cells after this time at concentrations of 1–3 *μ*M, in 0.3–4% serum-containing medium, and in 4% BSA-containing medium for 1–24 hours. After incubation the conditioned medium was either collected or discarded. Cells were washed twice with PBS and were removed from the culture surface with EDTA solution or with trypsin (PAA, Linz, Austria). The resulting cell suspension was given back to the collected conditioned medium, or it was collected separately from the conditioned medium in centrifuge tubes. The cell suspensions were centrifuged at 290 g, 4°C for 5 minutes, and the cell pellets were collected in 1 mL isoflow sneath. 

### 2.6. Flow Cytometry

Cell suspensions were examined in a Coulter EPICS XL-MCL (Vienna, Austria) by using the EXPO 32 software. Baseline conditions were measured by cells incubated without peptides. Increase of fluorescence signal related to the control baseline was measured by detecting either extra peaks in the histogram of the FL-1-channel or by shifting the histogram to the right compared to the control baseline [4]. Also peptide solutions were tested in the flow cytometer, and they did not show signals comparable with cell signals. 

### 2.7. Peptide-Coating Experiments

In order to investigate if HN-1 or other peptides might attach cells from suspension, glass cover slips were coated with peptides or with poly-D-Lysine (as a positive control) at 50 *μ*g/mL, as it was published before [16, 17]. After coating and washing steps, the cover slips were loaded with 1% BSA/medium at 37°C for 1 hour [18]. The cells were plated at 2 × 10^4^/mL in serum-free medium, for 1 hour at 37°C [18], followed by removal of medium with not adherent cells, 3 washes with serum-free medium, addition of serum-containing medium and culturing for 48 hours at 37°C. After culture, the cells were fixed in 4% PFA in PBS for 20 minutes, washed with PBS, stained routinely with Hematoxyllin, and covered with glass slides in glycerin-gelatin. The slides were visualized in an Olympus BX50 (Tokyo, Japan) microscope. 

### 2.8. Fluorescence Microscopy

SCC-25 cells were plated at 10^4^/0.5 mL in chambered slides and grown in culture medium for 48 hours, followed by medium replacement with 0.3% serum-containing medium and incubation with HN-1 or scr-HN1 at 1 *μ*M for 6 hours. The peptide-containing medium was removed and discarded, followed by washes with PBS, fixation in 4% Paraformaldehyde (PFA) for 20 minutes, DAPI-staining, and visualization in a fluorescent microscopy (Axio-ImageM, Zeiss, Jena, Germany) at 200x original magnification.

### 2.9. Statistical Data Analysis

Statistical significance of the difference between data sets was determined by one-way analysis of variance.

## 3. Results

### 3.1. Reaction of HN-1 Compared to Other Peptides with Cells in Published Conditions

**Figure 1:**
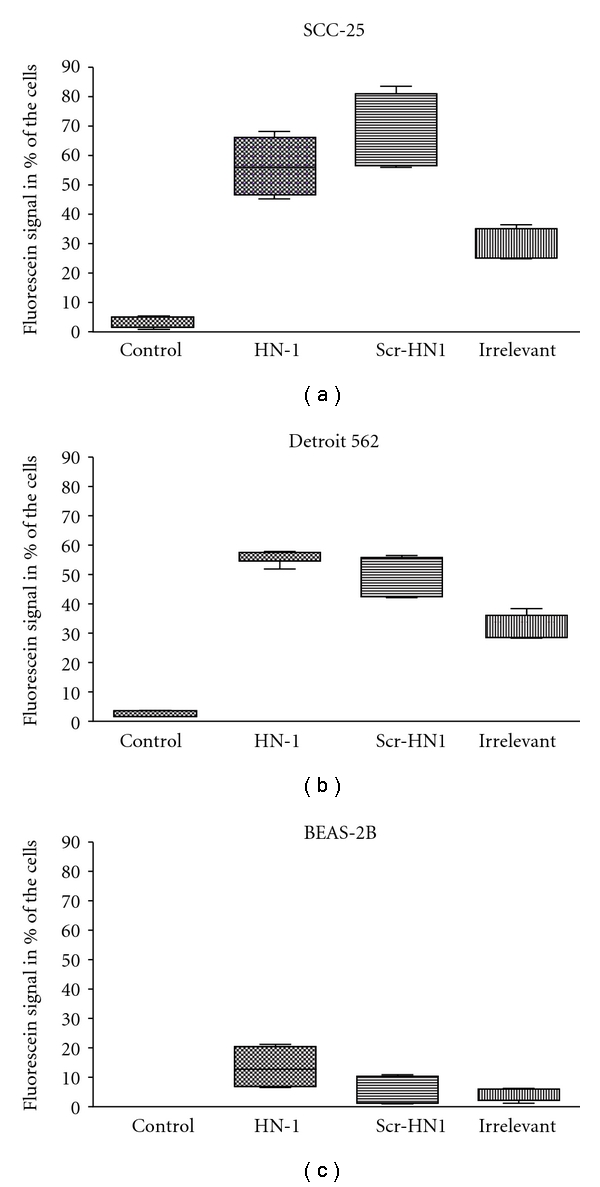
Peptide uptake in SCC-25 (a), Detroit 562 (b), and BEAS-2B (c) cells in published conditions. The cells were plated and incubated according to Bao et al. [4]. Tumor cells showed higher peptide interaction than BEAS-2B cells; the binding reaction to “irrelevant” peptide sequence was much lower than that of HN-1 and of its scrambled sequence. The highest binding was found in SCC-25 cells for scr-HN-1 peptide.

At the first step, we reproduced the experimental conditions of Bao et al. (2009) [4] and incubated the peptides at 3 *μ*M concentration with the cells for 24 hours. In these conditions, in average 56.15% of SCC-25 cells, 55.2% of Detroit 562 cells, and 12.92% of BEAS-2B cells showed FITC-positivity after incubation with HN-1; 69.2% of SCC-25 cells, 47.9% of Detroit 562 cells, and 5.45% of BEAS-2B cells showed FITC-positivity after incubation with scr-HN-1; 31.05% of SCC-25 cells, 31.6% of Detroit 562 cells, and 3.2% of BEAS-2B cells showed FITC-positivity after incubation with the “irrelevant peptide” (Figure 1). In general, the primary tumor- and the metastasis-originated cells showed higher peptide-related gain of fluorescence than the immortalized, normal epithelial BEAS-2B cells (Figure 1). Interestingly, this higher peptide-related gain of fluorescence was found by any peptide sequence. HN-1 reacted with SCC-25 and with Detroit 562 cells at the same level (56.15–69.2% positive cells in average, *P* > .05, One-way ANOVA), but scr-HN-1 significantly higher reacted with SCC-25 cells than with Detroit 562 cells (*P* = .005, One-way ANOVA) (Figure 1). 

### 3.2. Reaction of HN-1 Compared to Other Peptides with Cells during Shorter Incubation Time

3 *μ*M of HN-1 peptide represents ca. 4 mg/L peptide concentration, 24 hours incubation time is relatively suboptimal for therapeutic use, and 24 hours keeping the peptide intact in blood circulation conditions is unreliable. This might indicate a requirement of administration of an even higher peptide concentration. Seeing our concept of a targeting ligand for delivering a payload, lower peptide concentrations and shorter incubation times were foreseen. In this regard, SCC-25 cells were treated with HN-1, scr-HN1, and with the “irrelevant” peptide at 1 *μ*M peptides by 1 million cells for 1 h [3, 19]. In the first set, the peptide-containing medium was discarded after the 1 h incubation; the cells were washed three times with PBS and then removed from the culture surface by trypsinization. 2.3% of the SCC-25 cells showed fluorescein (FITC/FAM) reaction increased above the baseline, after 1h incubation with HN-1, and 2.9% after 1-h incubation with scr-HN-1. By increasing the concentration of the peptides to 3 *μ*M, 12.3% of the cells showed fluorescein (FITC/FAM) reaction increased above the baseline after incubation with HN-1, and 19.1% after incubation with scr-HN-1, and 2.5% after incubation with the “irrelevant peptide”. These results indicate that the peptide concentration cannot be decreased, and 1h incubation delivers drastically lower interaction with tumor or normal cells. 

In the second set, the peptide-containing medium was not discarded after the incubation, but collected; the cells were washed three times with PBS and then removed from the culture surface by trypsinization. Subsequently, the cells were given back to the corresponding peptide-conditioned medium; they were centrifuge-separated from the peptide solution; the pellet was dissolved and FACS-analyzed. By using 1 *μ*M peptide for 10^6^ plated cells, 84.7% of the cells showed fluorescein (FITC/FAM) reaction increased above the baseline after incubation with HN-1, 59.1% of the cells after incubation with scr-HN-1, and 38% of the cells after incubation with the “irrelevant” peptide. These results indicate that a double contact of the cells with the peptides dramatically improves their binding.

If BEAS-2B cells were used, the peptide-containing medium was not discarded after the incubation, the double contact of the cells with the peptides was allowed, 9.9% of the cells showed fluorescein (FITC/FAM) reaction increased above the baseline after incubation with HN-1, 34.1% of the cells after incubation with scr-HN-1, and 82.4% of the cells after incubation with the “irrelevant” peptide. 

These results were obtained with 0.3% serum-containing medium. With 4% serum-containing medium or with 4% serum albumin-containing medium, all cells were FITC-positive, indicating the complete binding of the peptide after the double contact. 

### 3.3. Analysis of Peptide Binding to Cells in Suspension

In previously described experiments, the giving back of peptide-containing medium to the cells incubated with the peptides before in an attached culture seriously increased the peptide interaction with the cells. This indicated that the cells might bind the peptides also in suspension, within the time from their removal from adherent culture, giving back of the peptide-containing medium, centrifugation, suspension in isotonic solution, and flow cytometry. This is not more than 30 minutes. 

In accordance, we have investigated if the cells in suspension bind the peptides within such a short time. We incubated 10^6^ SCC-25 cells with 1 *μ*M peptides in PBS or in 10% FBS-containing complete medium for 10–60 minutes at room temperature, mimicking the preparation conditions of trypsinized cells before flow cytometry. Importantly, the cells were washed after incubation. We determined the % of positive events, which showed emerged fluorescence levels from the baseline in the FL-1 channel by FACS. After 10 minutes, the controls showed 1.16 ± 0.06%, the incubation with HN-1 3.3 ± 0.3%, with scr-HN-1 3.5 ± 0.3, and with “irrelevant” 1.8 ± 0.2% positive events. After 60 minutes, the controls showed 0.6 ± 0.1%, the incubation with HN-1 2.3 ± 0.3%, with scr-HN-1 2.6 ± 0.3, and with “irrelevant” 0.5 ± 0.05% positive events. The usage of serum or albumin during the incubation did not improve these results (not shown). These results showed that the peptides were attached to less than 5% of the cells in suspension. For peptide-binding, a preincubation with peptide in attached culture was required. This means that there is no primary target for peptide-binding in suspended cells. An increased incubation time had no influence on the results.

**Figure 2:**
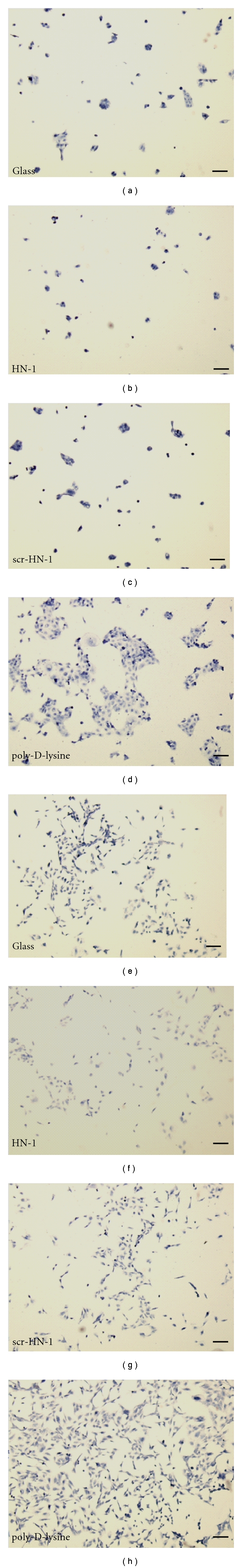
Attachment of SCC-25 (a)–(d) and BEAS-2B (e)–(h) cells to glass (a), (e), peptide-coated (b), (c), (f), (g) and poly-D-Lysine-coated (d), (h) glass surfaces. The cells were plated and incubated as described in Materials and Methods. After culture, the cells were fixed in 4% PFA in PBS for 20 minutes, washed, stained routinely with hematoxyllin, and covered with glycerin-gelatin. Images were taken at 40x original magnification. Bars: 100 *μ*m.

The previous results show that more contact of peptide to the cells improved its binding, but the peptide did not bind to the desired SCC-25 cells in suspension. This issue was tested in an alternative approach. We investigated if the peptides fixed to glass surface are suitable for binding tumor cells from a cell suspension, that is, if they can suit the concept of purging of circulating tumor cells of HNSCC. For this approach, we strictly followed already described methods [16, 17]. SCC-25 and BEAS-2B cells were given on the coated surfaces for 1 hour, then the surfaces were washed, and the cells were grown for two days. The SCC-25 cells showed relative inefficient growth on glass surface (Figure 2(a)); nevertheless, this was not improved, if the glass was coated by HN-1 or scr-HN-1 (Figures 2(b) and 2(c)). As a positive control poly-D-lysine coating was used (Figure 2(d)), which provided a surface of an efficient growth for SCC-25. BEAS-2B cells have grown efficiently on glass (Figure 2(e)), which has not been influenced by HN-1 or scr-HN-1 (Figures 2(f) and 2(g)); nevertheless, on poly-D-lysine coating these cells grew also better (Figure 2(h)).

### 3.4. Intracellular Peptide Analysis

**Figure 3:**
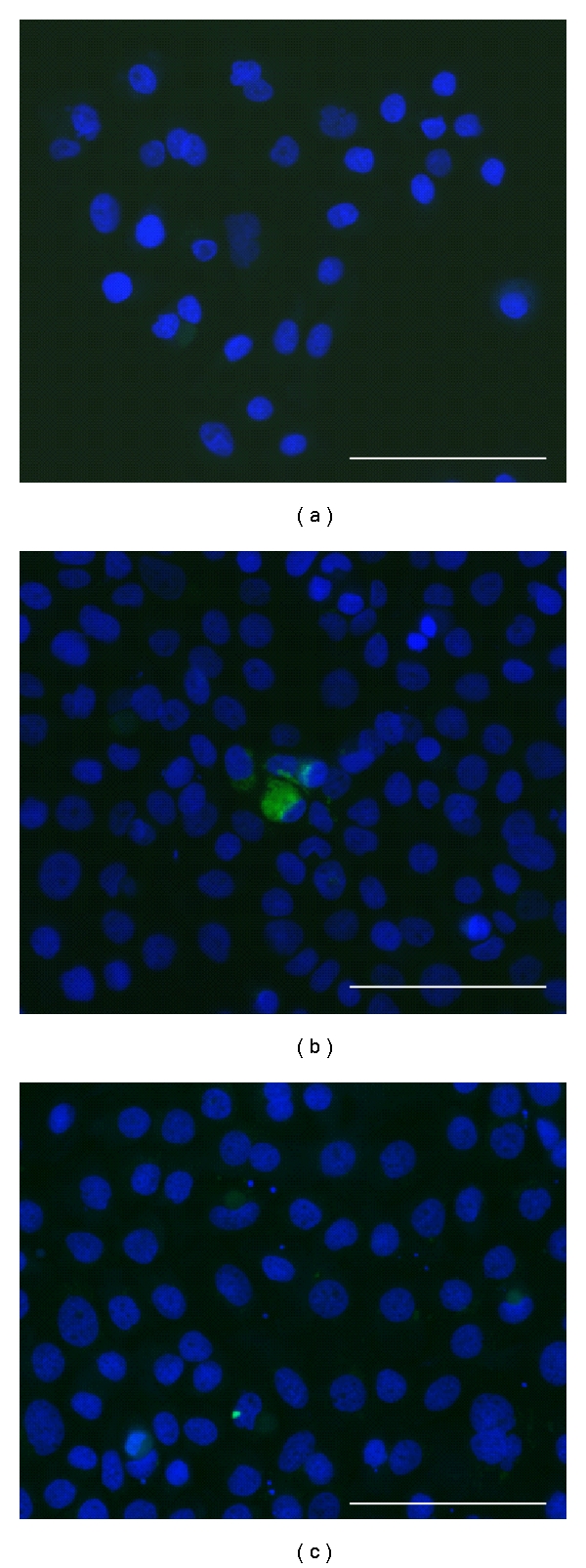
Internal peptide detection in SCC-25 cells after 6 hours. SCC-25 cells were plated at 10^4^/0.5 mL in chambered slides and incubated with HN-1 (b) and scr-HN-1 (c) for 6 hours, followed by washes with PBS, fixation in 4% PFA, DAPI-staining, and visualization in a fluorescent microscopy at 200x original magnification. (a) represents control cells. By the incubation with scr-HN-1 only very few cells showed intracellular localization, mainly at site of cell-cell adhesions. Bars: 100 *μ*m.

SCC-25 cells were treated with HN-1 or scr-HN-1 in 0.3% serum-containing medium for 6 hours; the cells were washed, fixed, and observed in a fluorescent microscope. Only very few cells showed internal fluorescein signal after incubation with HN-1; there were no FITC-positive cells after incubation with scr-HN-1 (Figure 3).

### 3.5. Structure and Folding Characteristics of the Used Peptides

**Figure 4:**
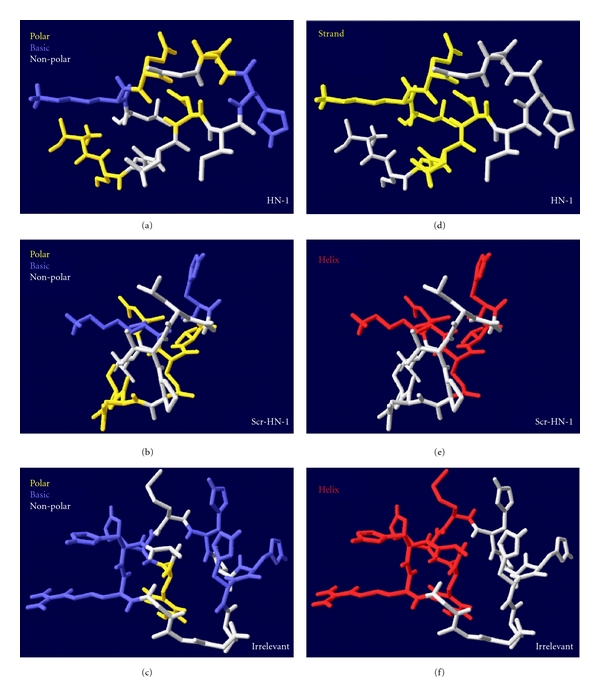
Three-dimensional peptides structure of the studied peptides. Representation of polar, basic, and nonpolar amino acids (a)–(c), and the secondary structure (d)–(f). The structure was calculated by the PEP-Fold software and visualized by Deepview/Swiss-Pdb-viewer. In (a)–(c) the polar, basic, and apolar amino acids are visualized in colors, in (d)–(f) the secondary structure elements are visualized. White color means “coil” in (d)–(f).

**Figure 5:**
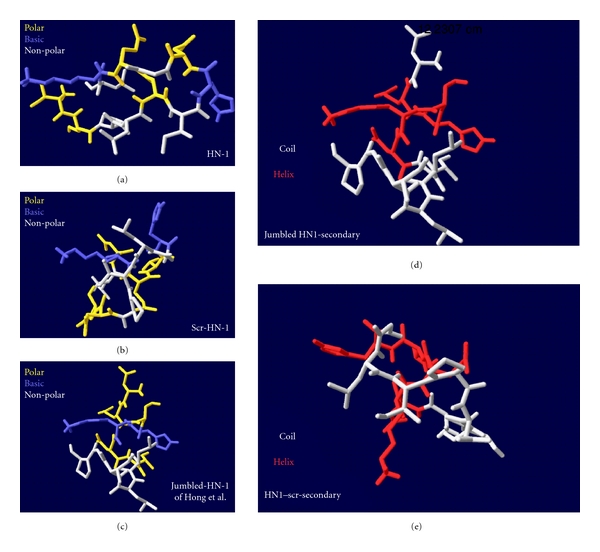
Comparison of HN-1 structure with different scrambled peptides. Representation of polar, basic, and nonpolar amino acids (a)–(c), and the secondary structure (d)–(e). The structure was calculated by the PEP-Fold software and visualized by Deepview/Swiss-Pdb-viewer. In (a)–(c), the polar, basic, and apolar amino acids are visualized in colors; in (d)–(e) the secondary structure elements are visualized.

By using the PEP-fold resource, we investigated the structure of the peptides. In the report of Hong and Clayman (2000) [1], an NQHSKNTLLIGP jumbling peptide was used, which in contrast to the original HN-1 (Table 2) did not internalize into squamous cell carcinoma cells. In the original HN-1, an intermittent sequence of polar and apolar amino acids was visible (**TS**PL**N**I**HN**G**QK**L) (the polar amino acids are labeled in bold). The polar amino acids are in single or double blocks interrupted by apolar amino acids. Hong and Clayman created a jumbled sequence [1], where they collected the polar amino acids together, and the apolar amino acids to the other side: **NQHSKNT**LLIGP. The three-dimensional structure of HN-1 is relative wide, based on the intermittent sequence of the polar and apolar amino acids (Figures 4(a) and 4(d)). The jumbled HN-1 (of [1]) is more compact, the basic amino acids are located in the middle of the molecule, in the same axis, nearly linearly (Figure 5(c)). In HN-1, the basic amino acids form an approximately 115° angle with each other (Figures 4(a) and 5(a)). In the jumbled HN-1, the apolar amino acids are located in the same side of the molecule (Figure 5(c)), while in HN-1 they are alternated (Figures 4(a) and 5(a)). We have generated a different scrambled sequence of HN-1: L**NKQTH**GLIP**NS** (the polar amino acids are labeled in bold). In this case, the polar and the apolar amino acids were clustered, but they were relative alternately represented in a polar-apolar-polar sequence. The three-dimensional structure of this peptide was also more compact than the original HN-1, the basic amino acids are perpendicular, and form an approximately 90° angle with each other, and the apolar amino acids are located alternated (Figures 4(b), 4(e), and 5(b)). The secondary structure motives of HN-1 and of the jumbled and scrambled peptides are also different. While HN-1 contains a strand (Figure 4(d)), both mixed sequences contain a helix (Figures 4(e) and 5(d)). In this study, a totally irrelevant peptide was also used as a control, called “irrelevant”, built form completely different amino acids, and showing different structures, with some accidental small similarities to HN-1 (Figures 4(c) and 4(f)). 

### 3.6. Amino Acid Sequence Analysis of HN-1

By analyzing of HN-1 sequence (TSPLN**IH**N**GQ**KL), the bold labeled amino acids have been found to be involved in protein-protein interactions using published analysis tools [15]. In the scr-HN1 peptide (**LN**K**QT**HGLIPNS), completely different amino acids were predicted to be involved in protein-protein interactions. Taking also these data into consideration Swiss-prot analysis of HN-1 and scr-HN1 was performed and no shared similar proteins were found. Based on the binding properties of HN-1 to HNSCC cells (binding exclusively to adherent culture of polarized cells), an HN-1 similar protein, hemicentin (fibulin-6), has been further investigated: TSP**LNIHN**GQ**K**L is the HN-1 sequence, where the bold labeled amino acids of HN-1 are identical with hemicentin. We have designed human Hemicentin primers, performed real-time RT-PCR, and no expression of hemicentin was found in HNSCC tumor cells and with HNSCC cells cocultured fibroblasts (not shown). 

## 4. Discussion

The HN-1 module is a 12-mer peptide, which was reported to preferentially bind and internalize into HNSCC cell lines in vitro; moreover, it was shown to be stable in vivo and able to localize into HNSCC xenograft tumors [1, 4]. Bao et al. [4] demonstrated the efficiency and selectivity of a HN1-PKC*ε* combined peptide in HNSCC cells in vitro, with a series of different FITC-labeled peptides (peptides at 3 *μ*mol/L, incubated 24–48 hours) followed by fluorescence-activated cell sorting (FACS) analysis. In normal epithelial cells, FITC-labeled HN1-PKC*ε* showed only uptake in 1.9% of the cells. In contrast, FITC-labeled HN1-PKC*ε* treatment of UMSCC1 and UMSCC36 cells resulted in 82.1% and 86.5% FITC-positive cells, respectively [4]. In our study, similar high uptake of HN-1 peptide was found in SCC-25 and Detroit 562 cells, and BEAS-2B showed significantly lower uptake (Figure 1). In this regard, our study confirmed these published results [1, 4] using other cell lines, which are commercially available from cell banks. These results support the following conclusions:

- HN-1 targets HNSCC tumor cells independently from that they are originated from primary tumor or from metastasis;
- the targeting of immortalized normal epithelial cells is significantly lower;
- a scrambled version of HN-1, where the sequence order of amino acids is different from the original HN-1, shows the same, not statistically different efficiency as HN-1 in the adapted conditions from Bao et al. [1, 4];
- the targeting of HN-1 is not strictly sequence specific; it might be related to the relative position angle of the positive-charged amino acids and to an intermittent structure of polar and apolar amino acids;
- normal, immortalized epithelial cells have taken any peptide with a lower efficiency.

In our study, we also tested other conditions, like other incubation times, concentrations, and serum conditions [2, 20]. We found that higher albumin and serum concentrations (4% instead of 0.3–1%) lead to increased peptide binding, and also to increased binding of unspecific peptides. It has been already reported that albumin influences the functions of peptides [21]. The peptide binding in short incubation time was seemingly efficient, (we could detect up to 100% of the cells showing gain of fluorescence), in contrast, it was only detectable with a “double contact” to the peptide, by keeping the peptide-containing medium and giving it back to the cells after resuspension with trypsin. PBS-washes could completely remove peptides from the cells. In this regard, we did not see strong and specific peptide binding on the cell surface within short incubation time, also washed slides did reveal very low internal peptide signals within the cells (Figure 3). 

Confirming the data of Bao et al., long incubation time (from 20 hours) and high peptide concentrations are required for effective binding and uptake. Bao et al. worked even with 30 *μ*M peptides [4]. Both the time and the concentration are suboptimal for further consideration. 

In our study, a strict sequence specificity of HN-1 was not confirmed in SCC-25 and Detroit 562 HNSCC cells. It seemed like that a relative position of positive-charged amino acids and an alternating position of polar and apolar amino acids are required for binding (Figures 4 and 5). Further outcome of our study is that, the target of HN-1 is not clear. Both the analysis of sequence specificity and the secondary structure analysis aimed to get nearer to the identification of the target. The sequence specificity could not be credited, while the used scrambled HN-1-sequence showing completely different sequence similarities than HN-1 showed similarly good uptake results as HN-1. Also the secondary structure identification could not bring us nearer to the identification of the target. The determination of protein-protein interactive amino acids also ruled out a defined interactive motive of HN-1, confirming the data of Hong that HN-1 probably does not bind through its NGQ-motive [1]. Nevertheless, some issues could have been claimed. The target, based on the sequence analysis and the experimental data, is involved in cell-matrix interaction or in cell-cell contact. It is required for the targeting that the cells grow in nodules and not separately distributed as BEAS-2B. Also the peptide uptake did not work in suspension, while it either requires the polar structure of the cells or the cell-cell contacts. HN-1 and scr-HN1 could not improve the attachment of the cells to glass surface; they do not interact with suspended cells. At the same time, it probably uses some attachment, adhesion, and growth properties of HNSCC, which supports its HNSCC-specificity. 

There are two critical points in the results of this study. The binding of HN-1 to normal (immortalized) epithelial cells is low, but it is not 0. It is also important to mention that those cells had lower binding to any peptide sequence in general (Figure 1). In the report of Hong and Clayman [1] cell lines from primary tumors of the floor of the mouth and the larynx were used. Bao et al. used malignant oral squamous cell carcinoma cells; we have used squamous cell carcinoma cells originated from primary lingual SCC, and metastatic SCC cells from the pharynx. This is the first report showing the efficiency of HN-1 in metastatic cells. The used cell lines represent differently staged original tumors and different histological locations. Taking also into consideration that squamous cell carcinoma of head and neck is usually a heterogeneous tumor, it is hard to expect that a 12-mer peptide effectively and sequence-specifically targets all HNSCC tumors. The in vivo xenograft results of Hong and Clayman [1] can be also explained by an affinity of the peptide to human tissue within the mouse; there is no direct proof for tumor specificity by those experiments. 

Targeted therapies represent an attractive approach to circumvent nonspecific cytotoxicity of conventional anticancer treatments [3]. Peptides do provide the advantage of small molecules, an advantage that enables them to serve as promising carrier molecules for drug targeting or as tracer molecules for tumor imaging. The technique for designing HNSCC-targeting peptides was comparable in several reports, a 12-amino-acid peptide phage display system [1, 3], but the cell lines used were always different, as also the amino acid sequence and secondary structure of the described peptides. Both studies, Hong and Clayman, 2000 and Nothelfer et al., 2009 [1, 3] generalize the findings based on in vivo data and state that the found peptide is HNSCC specific. Bao et al. used different cell lines and confirmed the findings of Hong and Clayman [4]. We could also see effective uptake of HN-1 in commercially available HNSCC cell lines. 

The main problem of HN-1 is that the target is not an identified receptor sequence, but a cell surface negative charged molecule, most probably a glycosaminoglycan-protein-complex. From the results, it seems that the presence of the target is dependent on the conditions. Interestingly, the change of amino acid sequence did not abolish the function of HN-1.

The sequence similarities of HN-1 to hemicentin were interesting, but hemicentin expression was not confirmed in HNSCC cells. Hemicentins are recently described extracellular matrix (ECM) proteins with a single ortholog in C. elegans that assembles into discrete tracks constricting broad regions of epithelial cell contact into adhesive and flexible line-shaped junctions. The pericellular localization of vertebrate hemicentins on epithelia and other cell surfaces suggests that vertebrate hemicentins, like their nematode counterpart, are secreted ECM proteins likely to have a role in the architecture of adhesive and flexible cell junctions, particularly in tissues subject to significant amounts of mechanical stress [22]. Taking the original concept of this study into consideration, HN-1 without clearly definable binding and uptake mechanisms is not a valid targeting ligand for nanoparticles carrying therapeutic payloads. Nevertheless, in combination with other peptides it might be still used for intended accumulation of adjuvant agents to tumor cells. 

## 5. Conclusions

Taken together, the results confirmed that peptide-targeting of a currently unknown cell surface structure in different HNSCC tumor cells of primary tumor and of metastasis is possible. The binding of HN-1-peptide is dependent on the experimental conditions and is not strictly structure specific. 

## Figures and Tables

**Table 1:** Cultured cells and culture conditions.

Name	Histology	Localization	Source	Culture medium	Purchased from
BEAS-2B	Epithelial cells	Bronchial	Not transformed immortalized epithelial	RPMI 1640	ECACC: European Collection Agency of Cell Cultures (Salisbury, UK),
SCC-25	Squamous cell carcinoma	Lingual	Primary tumor	Ham's F12 + Dulbecco's MEM (at 1 : 1)	DSMZ: German Collection of Microorganisms and cell cultures (Braunschweig, Germany)
Detroit-562	Squamous cell carcinoma	Pharyngeal	Metastatic	Ham's F12 + Dulbecco's MEM (at 1 : 1)	CLS: Cell Lines Service (Heidelberg, Deutschland)

All the cell lines were used within 6 months of purchase, and the identity was declared by the deliverers.

**Table 2:** Used peptides. The polar or charged amino acids were labeled in bold. Peptides were FITC-labeled at the NH2-terminal.

Peptide	Designed by	MW g /mol	Sequence1-letter-code	Sequence3-letter-code
HN-1	(Dr. Hong et al.)	1320,5	**TS**PL**N**I**HN**G**QK**L	Thr-Ser-Pro-Leu-Asn-Ile-His-Asn-Gly-Gln-Lys-Leu
HN-1-scr	Dr. F. Andreae, Graz, Österreich	1320,5	L**NKQTH**GLIP**NS**	Leu-Asn-Lys-Gln-Thr-His-Gly-Leu-Ile-Pro-Asn-Ser
Irrelevant	Storkbio, Tallin, Estonia	1550,7	GGG**RH**AY**H**M**H**P**HH**G	Gly-Gly-Gly-Arg-His-Ala-Tyr-His-Met-His-Pro-His-His-Gly

## Abbreviations

BSA: Bovine serum albumin
ECM: Extracellular matrix
EDTA: Ethylene-diamine tetraacetate
FACS;: Fluorescence administered cell sorting
FBS: Fetal bovine serum
FITC: Fluoresceinisothiocyanat
HNSCC: Head and neck squamous cell carcinoma
PBS: Phosphate buffered saline
Pdb: Protein data bank
PFA: Paraformaldehyd
PKCq: Proteinkinase Cq
SCC: Squamous cell carcinoma.
